# Bisphenol A and prostate cancer: from population exposure to context-dependent mechanisms and translational implications

**DOI:** 10.3389/fcell.2026.1949460

**Published:** 2026-09-30

**Authors:** Zhanhao Li, Hongliang Cao, Zihao Ye, Binbin Wang, Shuxin Li, Hao Wu, Zhiyuan Bai, Baoshan Gao

**Affiliations:** 1 Department of Urology II, The First Hospital of Jilin University, Changchun, China; 2 Department of Urology, Key Laboratory of Organ Regeneration and Transplantation of Ministry of Education, and National-Local Joint Engineering Laboratory of Animal Models for Human Diseases, The First Hospital of Jilin University, Changchun, China; 3 Department of Urology I, The First Hospital of Jilin University, Changchun, China

**Keywords:** androgen receptor signaling, bisphenol A, endocrine-disrupting chemicals, environmental exposure, epigenetic reprogramming, metabolic plasticity, prostate cancer

## Abstract

Bisphenol A (BPA) is a widespread endocrine-disrupting chemical with potential relevance to prostate cancer. This review provides a BPA-centered synthesis of evidence from human epidemiological studies, animal models, human prostate tissue and human-derived systems, and mechanistic investigations, while considering major BPA substitutes only where they inform substitution safety and class-informed assessment. Human studies provide limited and heterogeneous signals linking BPA exposure to prostate cancer incidence, prostate-specific antigen concentrations, and post-diagnosis outcomes, but causal inference is constrained by single-sample exposure assessment, cross-sectional or diagnosis-period designs, and heterogeneous endpoints. Experimental evidence supports a context-dependent model in which exposure timing, internal dose, hormonal milieu, androgen receptor status, cellular state, and subsequent biological challenge shape BPA-associated responses involving receptor signaling, epigenetic regulation, metabolism, redox homeostasis, and tissue organization. Rather than supporting uniform direct carcinogenicity, current evidence is more consistent with context-dependent modification of prostate biology, with distinct implications for cancer initiation, disease progression, hormone or treatment responsiveness, and biomarker alterations. These evidence domains should not be considered interchangeable. BPA measurements are not currently validated for prostate cancer screening, prognostic assessment, or treatment decisions. Progress will require repeated prospective biomonitoring, exposure-anchored human-relevant models, causal testing of proposed mediators, clinically verified outcomes, and targeted evaluation of major BPA substitutes such as bisphenol S (BPS) and bisphenol F (BPF), for which prostate-specific evidence remains comparatively limited.

## 1 Introduction

Prostate cancer (PCa) remains one of the most commonly diagnosed malignancies and a major cause of cancer-related mortality among men worldwide (Mottet et al., 2021; Rebello et al., 2021; Sung et al., 2021; Siegel et al., 2025). Current management ranges from active surveillance for selected low-risk localized disease to surgery, radiotherapy, and systemic treatment for recurrent or advanced disease, including androgen-deprivation therapy, androgen-receptor pathway inhibitors, chemotherapy, targeted therapies, and radioligand therapy (Huggins, 1942; Desai et al., 2021; Fizazi et al., 2023; Lowrance et al., 2023; Morgan et al., 2024; Garje et al., 2025). Despite these advances, progression to castration-resistant prostate cancer (CRPC) remains a major therapeutic challenge for patients with advanced disease (Gravis et al., 2018; Zhang et al., 2025). Prostate-specific antigen (PSA)-based screening also remains limited by imperfect specificity and the risks of overdiagnosis and overtreatment (Brawley et al., 2009; Daskivich et al., 2025; Monda et al., 2025). Alongside these clinical challenges, there is continuing interest in potentially modifiable environmental exposures that may influence prostate susceptibility or disease-related phenotypes.

Among environmental exposures of interest, endocrine-disrupting chemicals (EDCs) have received increasing attention. Bisphenol A (BPA), one of the most extensively studied EDCs, is widely used in polycarbonate plastics and epoxy resins, and human exposure occurs primarily through dietary ingestion, with additional contributions from inhalation and dermal contact (Vandenberg et al., 2007; Wetherill et al., 2007; Geens et al., 2012; Chen et al., 2016; Lv et al., 2017; Geueke et al., 2025). BPA is rapidly metabolized and eliminated after absorption (Völkel et al., 2002; Corrales et al., 2015; Kassotis et al., 2020); because exposure is frequent and recurrent, however, biomonitoring measurements may capture only a limited temporal window of internal exposure. Human studies have examined associations between BPA exposure and prostate cancer incidence, PSA concentrations, and post-diagnosis outcomes, but interpretation remains constrained by heterogeneous study designs, exposure assessments, biomonitoring time points, and clinical endpoints (Salamanca-Fernández et al., 2021; Li et al., 2024; Zhou et al., 2025).

Previous reviews have examined bisphenols across urological malignancies, broader effects of endocrine-disrupting chemicals on prostate biology, and the potential carcinogenic mechanisms of environmental endocrine disruptors (Pellerin et al., 2021; Lacouture et al., 2022; Feijó et al., 2025). More recently, a critical review of BPA and cancer epidemiology emphasized the limitations of short-term biomarkers, exposure misclassification, and the timing of biomonitoring relative to cancer development (Guha et al., 2026). These complementary reviews define the broader field but do not fully resolve an important cross-domain interpretive problem: population associations, animal studies, human prostate tissue studies, human-derived experimental systems, and mechanistic experiments differ substantially in their proximity to human disease and in the types of inference they can support. In particular, evidence concerning cancer initiation, disease progression, hormone or treatment responsiveness, and prostate-related biomarkers should not be treated as biologically or evidentially interchangeable.

Accordingly, this review is centered on BPA, for which the prostate-specific evidence base is substantially more developed than that for major substitutes. Bisphenol S (BPS), bisphenol F (BPF), and other BPA analogues are considered only where they inform substitution safety and class-informed assessment, and are not treated as having an evidence base equivalent to BPA. We integrate human epidemiological evidence, animal studies, human prostate tissue and human-derived models, and mechanistic findings within an explicit evidence hierarchy. Rather than assuming that BPA acts as a uniform, stand-alone prostate carcinogen, we evaluate how reported responses vary with exposure timing, internal dose, hormonal milieu, receptor status, cellular or disease state, subsequent biological challenge, and the endpoint examined. Particular emphasis is placed on distinguishing evidence relevant to cancer initiation or etiologic susceptibility, disease progression, hormone or treatment responsiveness, and biomarker alterations, and on defining where experimental observations do—and do not—support translational or clinical inference. Figure 1 summarizes the major routes of BPA exposure, phase II metabolism, and selected biological effects relevant to the scope of this review.

**FIGURE 1:**
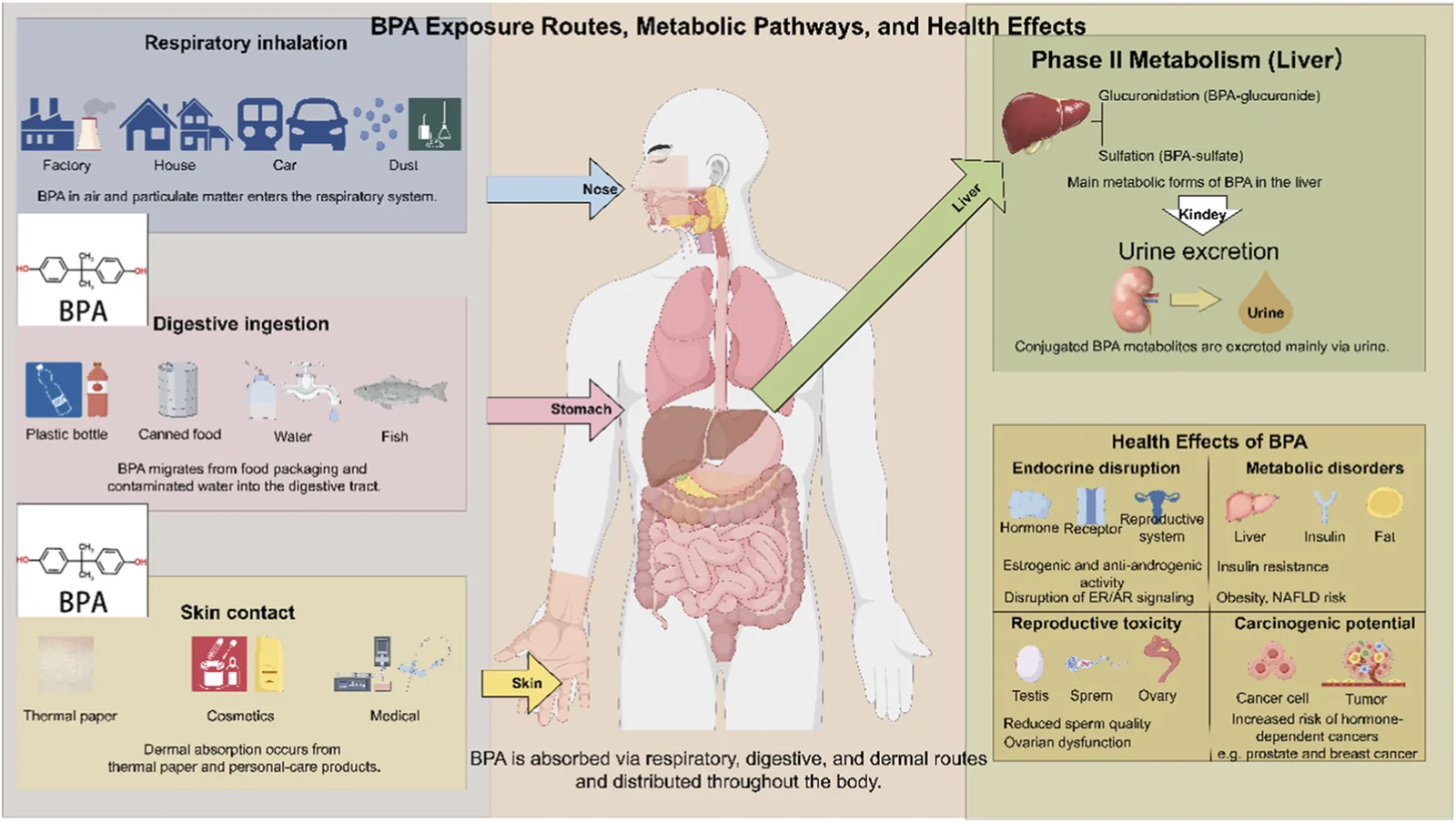
Exposure routes, metabolism, and selected biological effects of bisphenol A. BPA exposure occurs through multiple routes, including dietary ingestion, inhalation, and dermal contact. Following absorption, BPA undergoes phase II metabolism in the liver and is excreted primarily in urine. Repeated exposure can nevertheless result in recurrent internal exposure. BPA has been investigated in relation to endocrine, metabolic, reproductive, and cancer-related outcomes, while its relationship with prostate cancer remains incompletely defined. Abbreviations: BPA, bisphenol A; ER, estrogen receptor; AR, androgen receptor; NAFLD, non-alcoholic fatty liver disease.

## 2 Literature search, evidence selection, and interpretive framework

### 2.1 Search strategy and study selection

This review was conducted as a critical narrative synthesis informed by a structured literature search and literature update. PubMed and Web of Science Core Collection were searched from database inception through 5 September 2026 without restrictions on publication year. For the core BPA–prostate search, PubMed was searched using (“Bisphenol A” [MeSH] OR “bisphenol A” [Title/Abstract] OR “bisphenol-A” [Title/Abstract]) AND (“Prostatic Neoplasms” [MeSH] OR prostat*[Title/Abstract]). A separate analogue-focused PubMed search combined prostate-related terms with major BPA substitutes and related terms, including “bisphenol S,” “bisphenol F,” “bisphenol AF,” “bisphenol AP,” “bisphenol B,” “bisphenol analogue/analog,” and “BPA substitute/alternative.” Web of Science Core Collection was searched using Topic combinations of the corresponding BPA and analogue terms with prostat* to provide cross-database verification and to identify studies in which prostate relevance was represented in abstracts, author keywords, or indexing terms rather than explicitly in the title.

Peer-reviewed primary studies were considered for the core evidence synthesis when they directly measured BPA exposure or experimentally manipulated BPA and reported prostate cancer, prostate-related biomarkers, prostate pathology, findings in human prostate tissue or human-derived systems, or prostate-relevant cellular and mechanistic endpoints. Studies of BPA substitutes were included in the prostate-focused synthesis when they provided prostate-specific empirical evidence; broader analogue literature was used selectively only to contextualize substitution safety and evidence gaps. Studies outside male prostate or prostate-cancer biology were not included in the core prostate-specific evidence synthesis, although non-prostate studies could be used for general background on BPA exposure, toxicokinetic, or endocrine-disrupting properties. Reviews and other secondary sources were used primarily for background, conceptual framing, and citation tracing. Conference abstracts were used only for citation tracing and were not treated as standalone empirical evidence, while purely computational or network-based findings without empirical validation were considered hypothesis-generating rather than evidence of an established BPA-related mechanism.

Within the narrative synthesis, interpretive weight was assigned according to study design and proximity to human prostate disease rather than by applying a global numerical quality score. Particular consideration was given to prospective exposure assessment and clinically verified outcomes in human studies; exposure route, timing, dose, internal exposure information, subsequent biological challenge, and pathological endpoints in animal studies; and tissue origin, donor or disease state, receptor context, exposure duration, and functional endpoints in experimental models. Mechanistic studies were further interpreted according to whether the reported relationship was demonstrated directly after BPA exposure, established only in general prostate-cancer biology, or inferred through integration across different experimental systems. Because the objective was a critical narrative review rather than a systematic review or meta-analysis, no pooled effect estimates or formal global study-quality grading was performed. Studies were selected for detailed discussion based on relevance to prostate-specific outcomes, methodological transparency, and their contribution to evaluating evidence strength within the predefined framework.

### 2.2 Evidence hierarchy and definition of context-dependent susceptibility

Evidence was interpreted according to both the study design and the type of inference that it could support. First, human observational studies were considered capable of supporting associations and, when exposure assessment preceded diagnosis, temporal ordering, but not of independently establishing a causal mechanism. Second, BPA-specific experimental studies were considered evidence that BPA can produce a defined response within the tested model, dose, exposure window, hormonal environment, and cellular or disease state; such findings were not assumed to establish a causal effect in human prostate cancer. Third, studies of general prostate-cancer biology in which BPA was not tested were used to contextualize the functional relevance of a pathway, metabolic program, receptor state, or treatment context, but were not treated as evidence for a BPA-specific mechanism. Fourth, relationships assembled across separate studies or model systems were classified as integrative or model-based hypotheses unless the relevant causal sequence had been directly demonstrated experimentally.

Within this framework, “context-dependent modification of prostate susceptibility” does not denote a single disease endpoint. Rather, it describes variation in the direction or magnitude of BPA-associated responses according to exposure timing, internal dose, hormonal milieu, androgen-receptor status, cellular or disease state, subsequent biological challenge, and the endpoint examined. Disease-oriented evidence was therefore separated into four domains: cancer initiation or etiologic susceptibility, disease progression, hormone or treatment responsiveness, and biomarker alterations. Evidence in one domain was not considered evidence for another. For example, an association between BPA exposure and PSA concentrations does not demonstrate that BPA initiates prostate cancer, and altered treatment responsiveness in a selected experimental model does not establish an effect on cancer incidence.

Target-tissue detection and mechanistic or intermediate phenotypes were retained as supporting evidence categories rather than being automatically assigned to one of these four disease domains. Thus, detection of BPA in prostate tissue supports target-tissue accessibility but does not establish a biological effect, whereas changes in signaling, epigenetic regulation, metabolism, oxidative stress, centrosome organization, differentiation, or stromal remodeling may support biological plausibility without themselves demonstrating cancer initiation or progression. Human biomonitoring concentrations were also interpreted separately from nominal experimental concentrations; urinary or serum BPA measurements were not treated as numerically equivalent to concentrations applied directly to cultured cells.

## 3 Evidence linking BPA exposure to prostate cancer–related outcomes

### 3.1 Human epidemiological evidence

Human epidemiological evidence linking BPA exposure to prostate cancer-related outcomes remains limited and heterogeneous. Available studies differ substantially in design, timing, exposure assessment methods, and outcome definitions. These distinctions are important because evidence for incident prostate cancer, diagnosis-period associations, PSA alterations, and post-diagnosis progression addresses different biological and clinical questions and should not be interpreted interchangeably.

The strongest evidence for temporal ordering comes from the case–cohort study nested within the Spanish European Prospective Investigation into Cancer and Nutrition (EPIC-Spain). Among 575 incident prostate cancer cases and 1,772 male subcohort participants followed for a median of 16.9 years, serum BPA measured at recruitment was associated with increased prostate cancer risk in the first two exposure tertiles relative to concentrations below the limit of detection. However, the highest tertile was not statistically significant, and continuous and log-transformed exposure models were also non-significant (Salamanca-Fernández et al., 2021). Thus, although the prospective design establishes that BPA measurement preceded diagnosis, the categorical pattern does not support a simple monotonic exposure–response relationship. It should also not be interpreted as proof of a specific non-monotonic endocrine mechanism, particularly because exposure was assessed using a single baseline serum measurement.

Retrospective and diagnosis-period studies have reported additional associations, but they provide weaker evidence regarding temporality. In a Hong Kong case–control study, a questionnaire-derived cumulative BPA exposure index was positively associated with prostate cancer, although the exposure metric reconstructed long-term contact with BPA-containing food and beverage containers rather than directly measuring internal BPA concentrations (Tse et al., 2017). Three subsequent clinical studies measured urinary BPA during the diagnostic period and reported higher exposure measures among men with prostate cancer (Tarapore et al., 2014; Álvarez-González et al., 2024; Wang et al., 2024). Wang et al. observed a particularly large association in the highest BPA category, whereas Álvarez-González et al. reported a more modest association in a biomonitoring subset of only 60 participants. These studies therefore provide a recurring case–control signal, but most relied on a single urine sample obtained at or near diagnosis. Such measurements cannot determine whether the observed exposure preceded tumor initiation, and reverse causation cannot be excluded if existing disease or diagnosis-related changes affect exposure patterns, metabolism, or excretion.

Studies using PSA as the outcome require separate interpretation. In an analysis of 2,768 male participants from the National Health and Nutrition Examination Survey (NHANES) 2003–2012, urinary BPA was positively associated with serum PSA after multivariable adjustment, and a non-linear relationship was reported when PSA was analyzed as a continuous outcome (Li et al., 2024). However, the adjusted non-linear association was not evident when PSA was dichotomized. More importantly, PSA elevation is a biomarker alteration rather than a clinically verified prostate cancer endpoint. Accordingly, this study does not demonstrate that BPA increases prostate cancer incidence, and the reported concentration around which the spline relationship changed should not be interpreted as a validated BPA threshold for prostate cancer screening or risk stratification.

Evidence for post-diagnosis progression is more preliminary. Zhou et al. reported that higher urinary BPA was associated with shorter biochemical recurrence-free survival in a prospective cohort of patients undergoing radical prostatectomy, together with survival-related associations in secondary analyses of NHANES participants with prostate cancer (Zhou et al., 2025). These observations extend the epidemiological literature from cancer occurrence to progression-related outcomes. However, the clinical populations with BPA and outcome data were relatively small, BPA was assessed at a single time point, information on multivariable clinical adjustment was limited in the main report, and the findings have not yet been independently replicated. They are therefore better regarded as preliminary evidence of a possible progression-related association than as validation of BPA as a prognostic biomarker.

Several exposure-assessment issues further limit comparisons across the human literature. The studies used serum BPA, spot urinary BPA, creatinine-normalized urinary BPA, or questionnaire-derived cumulative exposure indices, which represent different dimensions of exposure and cannot be treated as equivalent measures of internal dose. Creatinine normalization reduces variability attributable to urine dilution, but single spot urinary BPA measurements remain subject to substantial within-person temporal variability (Ye X. et al., 2011; Morgan et al., 2018). Renal function, diet, occupation, lifestyle factors, and co-exposures to other environmental chemicals were also not assessed or adjusted for consistently across studies. These limitations are particularly important for diagnosis-period studies, in which exposure measurement and disease status are temporally close.

Overall, current human evidence suggests associations between BPA exposure and several prostate cancer-related outcomes, but it does not establish a causal relationship with prostate cancer initiation. The prospective EPIC study provides the strongest temporal evidence, whereas most other positive findings derive from diagnosis-period case–control studies, cross-sectional biomarker analyses, or small post-diagnosis cohorts. Future studies will require repeated pre-diagnostic biomonitoring, clinically verified outcomes, improved characterization of relevant exposure windows, and more complete assessment of hormonal, renal, lifestyle, and co-exposure factors to determine whether BPA contributes to prostate cancer risk or progression in defined biological contexts. The principal human studies and their methodological constraints are summarized in Table 1.

**TABLE 1:** Human studies of BPA exposure and prostate cancer-related outcomes.

Study	Design, population, and outcome domain	BPA assessment: matrix, timing, and method	LOD/LOQ and normalization	Main adjusted covariates	Main effect estimate	Methodological considerations
Salamanca-Fernández et al., 2021	EPIC-Spain prospective case–cohort; 575 incident PCa cases and 1,772 male subcohort participants; median follow-up 16.9 years. Domain: Cancer initiation/etiologic susceptibility	Single baseline serum total BPA collected before diagnosis; enzymatic deconjugation followed by DLLME–UHPLC-MS/MS	LOD 0.2 ng/mL; <LOD assigned LOD/√2; urine-dilution normalization N/A	Cox models stratified by center and age group; adjusted for age, education, BMI, physical activity, smoking, alcohol	Vs. < LOD: T1 HR 1.40 (1.05–1.88); T2 1.37 (1.02–1.82); T3 1.31 (0.98–1.74); p-trend = 0.069. Continuous and log2 models NS.	Strongest temporal design, but one baseline sample for a non-persistent chemical; no stable monotonic exposure–response; residual confounding and unmeasured mixtures remain possible
Tse et al., 2017	Hospital-based case–control; 431 histologically confirmed newly diagnosed cases, 402 hospital controls. Domain: Etiologic association	No biomonitoring. Questionnaire-derived cumulative BPA exposure index (CBPAI), based mainly on lifetime use of food/beverage containers, exposure intensity, frequency, and duration	N/A	Full model: Age, marital status, unemployment, family PCa history, deep-fried food, pickled vegetables, green tea, night-shift work, CBPAI	Middle vs. low OR 1.54 (1.05–2.26); high vs. low OR 1.57 (1.01–2.44); adjusted p-trend = 0.057	No measured internal dose; recall and exposure misclassification; hospital controls; predominantly ingestion-based index; adjusted trend did not reach conventional statistical significance. Authors acknowledge need for validation against repeated biomonitoring
Tarapore et al., 2014	Biopsy-based clinical study; 27 PCa cases, 33 non-PCa patients. Domain: Diagnosis-period association	Single pre-biopsy spot urine; total BPA by HPLC-ESI-MS/MS after enzymatic hydrolysis	MDL 0.05 ng/mL; <MDL assigned MDL/√2; creatinine-normalized	Fixed-effect model controlled age category and serum PSA; PCa status was used as an explanatory variable for LnBPA rather than estimating a conventional PCa OR	Creatinine-adjusted BPA geometric mean 5.74 μg/g (95% CI 2.63–12.51) in PCa vs. 1.43 μg/g (0.70–2.88) in non-PCa, p = 0.012	Very small sample; cases were older than non-PCa participants; one diagnosis-period urine sample; no conventional adjusted PCa odds ratio; temporality and reverse causation remain unresolved. These data should not be interpreted as evidence of etiologic risk, causality, or validated diagnostic/prognostic utility
Wang et al., 2024	Hospital case–control; methods report 187 biopsy-confirmed PCa cases and 151 controls. Domain: Diagnosis-period association	Single morning urine collected from treatment-naïve cases and controls; SPE–UPLC-MS/MS; multiple bisphenols/phthalates measured	Bisphenol LOD range 0.02–0.12 ng/mL; BPA-specific LOQ not stated in main article; creatinine-normalized	Age, BMI, marital status, education, smoking, alcohol	BPA 0.45–<1.43 vs. < 0.45 μg/g Cr: adjusted OR 2.87 (1.16–7.12); ≥1.43 vs. < 0.45: 7.33 (2.63–20.43)	Single diagnosis-period sample; wide CI; residual co-exposure possible; control selection; internal discrepancy in case count (187 in methods vs. 189 in tables)
Álvarez-González et al., 2024	Biopsy-based study, 150 cases/150 controls; urinary biomonitoring only in 30 cases + 30 controls. Domain: Diagnosis-period association	Single urine sample during diagnostic period; enzymatic hydrolysis + liquid–liquid microextraction + GC-MS.	BPA-specific LOD/LOQ: NR in main article; urinary dilution normalization: NR; concentration unit for Table 5 in the source article: NR.	Subsample models incorporated PSA ≥10 ng/mL, physical exercise, and measured pollutants BaP, BPA, and ethyl-paraben	BPA: Full model OR 1.32 (1.09–1.59); backward-selection model OR 1.28 (1.09–1.51)	Very small biomonitoring subset; exposure measured contemporaneously with diagnosis; no longitudinal exposure assessment; authors explicitly state absence of temporal concordance and cumulative-exposure information
Li et al., 2024	NHANES 2003–2012 cross-sectional analysis; 2,768 men. Domain: Biomarker alteration, not PCa incidence	Urinary BPA from NHANES; serum total PSA was the actual outcome. Assay details were referred to NHANES resources rather than reported in the article	BPA assay LOD/LOQ: NR in article; continuous BPA modeled as BPA/creatinine (ng/mg)	Race/ethnicity, age, PIR, education, marital status, cholesterol, HDL, HbA1c, BMI, diabetes, hypertension, tumor history, smoking, drinking	Fully adjusted continuous association: PSA +2.760 ng/mL (95% CI 1.506–4.014); Q4 vs. Q1: +1.074 ng/mL (0.862–1.286)	PSA ≥4 ng/mL was labeled “high cancer risk” by authors but is not confirmed PCa. Cross-sectional, single-sample exposure; residual confounding and within-person variability. The reported 4.46 ng/mL spline value is not a clinical BPA threshold
Zhou et al., 2025	Prospective post-prostatectomy WCHPCa cohort plus secondary NHANES PCa survival analyses. Domain: Disease progression/post-diagnosis outcome	Pretreatment urine in WCHPCa; HPLC-MS/MS after SPE; urinary BPA in both cohorts normalized to creatinine. BCR defined as two consecutive postoperative PSA values ≥ 0.2 ng/mL	LOD/LOQ: NR in main article; creatinine-normalized	Clinical multivariable covariate adjustment for the BPA survival analyses is NR in the main article; methods describe Kaplan–Meier/log-rank analysis	Fig. 2 reports WCHPCa HR 4.21 (95% CI 1.33–13.29), short-term NHANES HR 2.40 (1.16–4.97), and long-term NHANES HR 1.83 (1.05–3.21); corresponding p values 0.0077, 0.02, and 0.02	Small cohorts, single exposure assessment, limited information on clinical covariate adjustment in main article, no independent replication. Authors themselves acknowledge limited statistical power/generalizability

Abbreviations: BPA, bisphenol A; BCR, biochemical recurrence; CBPAI, cumulative BPA exposure index; CI, confidence interval; Cr, creatinine; DLLME, dispersive liquid–liquid microextraction; GC-MS, gas chromatography–mass spectrometry; HR, hazard ratio; LOD, limit of detection; LOQ, limit of quantification; MDL, minimum detection limit; NHANES, national health and nutrition examination survey; NR, not reported in the main article; OR, odds ratio; PCa, prostate cancer; PSA, prostate-specific antigen; SPE, solid-phase extraction; UHPLC/UPLC-MS/MS, ultra-high/ultra-performance liquid chromatography–tandem mass spectrometry. NR indicates information not reported in the main publication and should not be interpreted as evidence that the procedure was not performed.

### 3.2 Evidence from animal models

Animal studies indicate that BPA can alter prostate phenotypes, but the outcomes vary with developmental timing, exposure route and internal dose, subsequent biological challenge, and the endpoint examined. Developmental BPA exposure has been associated with persistent changes in prostate growth and histology in mice, although these findings were not neoplastic endpoints (Nagel et al., 1997; Taylor et al., 2020).

The clearest positive pattern for neoplasia-related susceptibility comes from Sprague–Dawley rat models in which early-life BPA exposure was followed by testosterone-plus-estradiol (T + E) treatment in adulthood. Neonatal BPA alone did not induce prostatic intraepithelial neoplasia (PIN) in the Ho et al. model, whereas subsequent T + E exposure markedly increased PIN susceptibility (Ho et al., 2006). Prins et al. further showed that oral and subcutaneous administration produced different internal BPA concentrations despite the same administered dose, emphasizing the importance of route and pharmacokinetics (Prins et al., 2011). Later studies identified lobe- and dose-specific increases in PIN severity, microinvasion, or tumor multiplicity after T + E, but did not demonstrate a consistent increase in adenocarcinoma incidence; related stem/progenitor changes represent cellular susceptibility phenotypes rather than neoplasia (Prins et al., 2017; Prins et al., 2018). These studies therefore support altered responsiveness to a later hormonal challenge rather than BPA acting as a sufficient tumor-initiating exposure.

Other experimental paradigms produced different outcomes. In the Consortium Linking Academic and Regulatory Insights on the Toxicity of Bisphenol A (CLARITY-BPA) core study, oral BPA exposure without a later hormonal challenge did not produce a consistent prostate pathology pattern (Camacho et al., 2019). Maternal or perinatal BPA exposure also did not enhance prostate carcinogenesis in the 3,2′-dimethyl-4-aminobiphenyl (DMAB) or N-methyl-N-nitrosourea (MNU)-plus-testosterone models (Ichihara et al., 2003; Bosland et al., 2023). These findings should not be treated as simple contradictions of the T + E studies because the later biological challenges, exposure conditions, and pathological endpoints differ substantially across models.

Adult studies place BPA-associated prostate phenotypes within distinct hormonal, dietary, and pre-existing tissue contexts rather than establishing a general effect-modification rule. BPA-associated hyperplasia, inflammation, low-grade PIN, or prostate enlargement has been reported in Noble rats, gerbils, and older beagle dogs, but these studies were limited by small groups, model-specific pathology, exposure conditions, or the absence of carcinoma endpoints (Lam et al., 2016; Facina et al., 2018; Wang et al., 2022). In particular, the Facina study does not establish a BPA–high-fat-diet interaction because a formal interaction test was not reported.

Overall, the animal evidence does not support a uniform, stand-alone prostate carcinogenic effect of BPA. The most consistently reported positive pattern within the available literature is developmental BPA exposure followed by a later T + E challenge, although much of this evidence derives from closely related Sprague–Dawley challenge paradigms and therefore does not constitute broad independent replication. BPA exposure without such a challenge, and studies using DMAB or MNU-plus-testosterone, have not reproduced the same lesion pattern. Differences in exposure window and route, internal dose, strain, later biological challenge, prostate region, and pathological criteria remain central to interpretation and limit extrapolation to human prostate cancer. The principal animal models, exposure conditions, pathological endpoints, and evidential boundaries are summarized in Table 2.

**TABLE 2:** Key animal studies of BPA exposure and prostate-related outcomes, organized by exposure window, internal-exposure characterization, later biological challenge, pathological endpoint, and evidential boundary.

Study	Animal design, sex/age, and sample size	BPA exposure: window, route, dose/vehicle, and internal exposure	Later context	Endpoint and pathology conduct/blinding	Result and statistical status	Evidence boundary
Nagel et al., 1997	CF-1 mice; male offspring; adult endpoint at 6 months; BPA groups n = 7; pooled controls n = 11 (5 unhandled +6 corn-oil vehicle controls)	Maternal oral micropipette feeding, GD11-17; 2 or 20 μg/kg/day; tocopherol-stripped corn oil. Internal BPA: NR.	None	Adult prostate weight; blinding: NR.	Both BPA doses increased prostate weight by 30% and 35%, respectively (p < 0.01)	Small developmental morphology study; prostate weight is not a neoplastic endpoint
Ho et al., 2006	Sprague‐Dawley rats; male neonates; 20–30 pups per neonatal treatment, subsequently divided between adult treatments; analyzed n per endpoint NR	SC on PND1, 3, and 5; 10 μg/kg per administration; tocopherol-stripped corn oil. Internal BPA: NR.	Adult T + E implants from PND90 for 16 weeks; empty-implant controls	Dorsal/lateral/ventral prostate PIN, hyperplasia, inflammation; sections coded and scored blinded	BPA alone did not induce PIN. With T + E, PIN incidence was 100% versus 40% in neonatal vehicle controls; PIN score increased from 0.52 to 1.3 (P < 0.01)	Direct evidence for enhanced PIN susceptibility in this specific neonatal SC plus adult T + E model; not evidence of BPA-alone carcinogenesis
Prins et al., 2011	Hsd:SD rats; male neonates; pathology cohort 15–25 pups/group. Separate pharmacokinetic study: 180 male pups; 90 SC and matched oral exposure	Oral or SC on PND1, 3, and 5; 10 μg/kg per administration; 0.02% ethanol in tocopherol-stripped corn oil. Serum free/total BPA measured by HPLC-MS/MS (LOD 0.05 ng/mL)	Adult T + E from PND90 for 16 weeks	Ventral, lateral, and dorsal prostate PIN/hyperplasia/inflammation; sections coded and scored blinded	Free/total BPA Cmax values were 1.77/2.00 ng/mL after SC and 0.26/1.02 ng/mL after oral exposure. Both routes increased PIN scores in the ventral and lateral lobes (P < 0.05); lateral-lobe PIN incidence was 100% (SC) and 90% (oral) versus 64% in controls (P < 0.02)	Demonstrates route-dependent neonatal pharmacokinetics and a T + E-challenge phenotype; administered dose alone is not an internal-dose surrogate
Prins et al., 2017	Sprague‐Dawley rats; male neonates. Day-200 pathology: n = 8–11/group; 1-year carcinoma analysis pooled two cohorts (n = 14–21/group, depending on endpoint)	SC on PND1, 3, and 5; 0.1–5,000 μg/kg; tocopherol-stripped corn-oil vehicle. PND3 serum free BPA/BPA‐glucuronide measured by UHPLC-MS/MS; free BPA quantifiable from 10 μg/kg	With or without adult T + E from PND90; 4 months (day-200 cohort) or to 1 year (aging cohort)	Lobe-specific PIN, hyperplasia, inflammation, microinvasion, and adenocarcinoma; coded, blinded pathology; uncertain diagnoses resolved by consensus	Without T + E, no significant BPA-related pathology at day 200. With T + E, PIN responses were lobe- and dose-specific. At 1 year, 10 μg/kg BPA increased lateral-lobe microinvasion; glandular carcinoma incidence was not significantly increased	Supports lobe-, dose-, and challenge-specific susceptibility. Focal carcinoma observations do not establish a uniform carcinogenic response
Prins et al., 2018 - pathology cohort	NCTR‐Sprague‐Dawley rats; males. T + E Set 3 analyzed n: Vehicle 16, EE 17, BPA 2.5 μg/kg 6, 250 μg/kg 12, 25,000 μg/kg 9; 25 and 2,500 μg/kg groups (n = 4 each) not statistically analyzed	Maternal/pup oral gavage GD6-PND21; 2.5–25,000 μg/kg/day; 0.3% aqueous CMC. Internal BPA in this pathology cohort: NR.	Adult T + E implants at PND90 to 1 year	Lateral-lobe PIN severity and periurethral DLP ductal adenocarcinoma; prostate histopathology blinded	PIN incidence was similar, but selected BPA doses increased PIN severity. At 2.5 μg/kg/day, tumor multiplicity among tumor-bearing animals increased four-fold (p < 0.001); adenocarcinoma incidence did not differ significantly	Supports challenge-contingent changes in lesion severity and tumor multiplicity at selected doses; the absence of an increase in adenocarcinoma incidence limits inference regarding tumor occurrence
Prins et al., 2018 - stem/progenitor cohort	NCTR‐Sprague‐Dawley rats; males; prostasphere analyses n = 3–5/group (one 25 μg/kg/day group reduced to n = 3 after technical loss)	Continuous oral gavage GD6 to 6 months; 2.5, 25, or 250 μg/kg/day; 0.3% aqueous CMC. Internal BPA: NR.	No carcinogenic hormonal challenge	DLP stem/progenitor number, prostasphere size, and lineage-related markers; not a pathology study	2.5 μg/kg/day increased prostasphere number; 25 and 250 μg/kg/day altered progenitor proliferation and lineage-related markers, with selected significant comparisons	Cellular homeostasis endpoint only; altered stem/progenitor phenotypes are not neoplasia or established causal mediators
Camacho et al., 2019	NCTR‐Sprague‐Dawley rats; both sexes; male prostate denominators varied by arm, sacrifice, and endpoint (approximately 20–26 at interim and up to 50 at terminal sacrifice)	Maternal/pup oral gavage GD6-PND21 (stop-dose) or continued to 1–2 years; 2.5–25,000 μg/kg/day; 0.3% aqueous CMC. Separate PND4/21 d^6^-BPA dosimetry cohorts measured serum unconjugated and total BPA	None; stop-dose and continuous-exposure arms	Systemic pathology including dorsal/lateral prostate; standardized criteria; independent QA and a treatment-blinded pathology working group reviewed reproductive organs	No consistent BPA-related prostate pathology pattern across dose/exposure arms. Isolated significant inflammatory findings did not form a coherent dose- or time-related pattern	No consistent BPA-related prostate pathology signal was identified under this no-challenge design; this study does not directly test susceptibility to a later hormonal challenge
Ichihara et al., 2003	F344 rats; male F1 offspring; 21/group allocated to DMAB arms and 12/group to non-DMAB arms; evaluable pathology n = 15–19/group in DMAB arms and n = 12/group in non-DMAB arms	Maternal intragastric gavage through gestation and lactation; 0.05, 7.5, 30, or 120 mg/kg/day; 0.5% CMC‐Na. Internal BPA: NR.	At 5 weeks: DMAB 50 mg/kg SC, 10 injections at 2-week intervals; corn-oil controls	PIN and carcinoma in ventral, dorsolateral, and anterior prostate; pathology blinding: NR.	No BPA-related difference in DMAB-induced PIN or carcinoma incidence; no significant pairwise differences reported	Negative result in a transplacental/lactational oral-exposure plus chemical-carcinogen model; DMAB is not interchangeable with T + E
Bosland et al., 2023	Sprague‐Dawley rats; male offspring; effective n = 31 control, 21 low-dose, and 26 high-dose after excluding animals that died before day 200 after MNU or lost testosterone implants	Maternal Alzet minipump exposure GD9-PND28; 2.5 or 25 μg/kg/day. Vehicle: NR in the main article. Present-study offspring serum/tissue BPA: Not measured; separate dam data are not a substitute	At 10–12 weeks: Flutamide, testosterone propionate, MNU 30 mg/kg IP, then chronic testosterone implant; followed 57–58 weeks after MNU	Dorsolateral/anterior prostate and accessory sex-gland carcinomas; pathology blinding: NR.	Prostate carcinoma incidence: 40% control, 33% low BPA, 23% high BPA; neither BPA group differed significantly from control	Negative evidence for enhancement in this MNU-plus-testosterone model; absence of present-study internal-exposure measurement limits exposure interpretation
Lam et al., 2016	Adult Noble rats; 16 weeks at treatment start; n = 3–4/group	32-week SC SILASTIC implants: 15 or 320 mg BPA plus separate 30-mg testosterone implants; empty-capsule controls. Serum free BPA measured by HPLC-MS/MS: 1.06 and 10.37 ng/mL in low/high BPA groups	Physiological testosterone replacement; T + E2 and T-only comparators	Lateral/ventral/dorsal/anterior prostate weight, hyperplasia, PIN, inflammation, and transcriptomics; PIN scored blinded	Low/high BPA groups showed increased prostate weight, hyperplasia, low-grade PIN, and lymphocyte infiltration; selected comparisons significant as reported	Adult hormonal-context model with measured serum BPA, but very small groups and implant delivery limit inference about cancer initiation or oral exposure
Facina et al., 2018	Adult gerbils, 100 days at start; males; n = 6/group	Six months of BPA in drinking water, target 50 μg/kg/day; BPA mixed with ethanol then diluted in water. Internal BPA: NR.	Control or high-fat/high-calorie diet.	Ventral and dorsolateral lesions, inflammation, and immunohistochemical markers; pathology blinding: NR.	BPA, diet, and BPA + diet groups had lesion/inflammation differences. Significant lesion-multiplicity findings were lobe- and treatment-specific; no formal interaction test established synergy	Supports model-bounded, lobe-specific lesion associations in adult gerbils; the absence of a formal interaction test does not support a claim of BPA–high-fat-diet synergy
Wang et al., 2022	Adult male beagle dogs >5 years with prostate volume ≥21 cm^3^; n = 6/group (molecular subsets n = 4)	Daily oral administration mixed with feed for 8 weeks; 2, 6, or 18 μg/kg/day. Vehicle: Reported but composition NR in the main article. Internal BPA: NR.	Pre-existing prostate enlargement; no carcinogenic challenge	Prostate volume, organ coefficient, epithelial height, and hyperplastic morphology; pathology blinding: NR.	Dose groups increased prostate volume and epithelial height; higher-dose organ coefficient and selected molecular/hormonal measures were significant as reported	Short-term BPH-like phenotype in a pre-existing enlarged-prostate context; no carcinoma endpoint or evidence of tumor induction
Taylor et al., 2020	CD-1 mice; male offspring. Initial group n: Control-empty 19, BPA-empty 16, control-T + E2 22, BPA-T + E2 13; survival and endpoint-specific n varied	Maternal oral micropipette dosing GD11-17 plus pup oral dosing PND1-15; 20 μg/kg/day; tocopherol-stripped corn oil. Fetal free BPA exposure cited from prior pharmacokinetic work; no present-study BPA assay	At 10 weeks, T + E2 or empty silastic capsules for 4 months	Prostate size, urinary obstruction-associated urogenital pathology, and selected histology; necropsy, grading, and histology were blinded	Perinatal BPA increased adult prostate-size scores (p < 0.05). Hyperplasia/prostatitis were described in obstructed T + E2-exposed animals; group-level neoplastic endpoints were not assessed	Non-neoplastic mouse urogenital model. Obstruction-associated pathology cannot be interpreted as carcinogenesis

NR indicates information not reported in the main publication and should not be interpreted as evidence that the procedure was not performed. Statistical significance refers only to the specific comparison reported in the source study; numerical differences without a reported statistical test were not treated as statistically significant. Nominal administered dose should not be interpreted as equivalent to measured internal exposure or to BPA concentrations measured in human biological matrices.

Abbreviations: BPA, bisphenol A; BPH, benign prostatic hyperplasia; CMC, carboxymethyl cellulose; CMC-Na, sodium carboxymethyl cellulose; Cmax, maximum observed concentration; d6-BPA, deuterated bisphenol A; DLP, dorsolateral prostate; DMAB, 3,2′-dimethyl-4-aminobiphenyl; E2, 17β-estradiol; EE, ethinyl estradiol; GD, gestational day; HPLC-MS/MS, high-performance liquid chromatography–tandem mass spectrometry; IP, intraperitoneal; LOD, limit of detection; MNU, N-methyl-N-nitrosourea; n, number of animals or samples analyzed; NCTR, National Center for Toxicological Research; NR, not reported in the main article; PIN, prostatic intraepithelial neoplasia; PND, postnatal day; QA, quality assurance; SC, subcutaneous; T, testosterone; T + E, testosterone plus estradiol; T + E2, testosterone plus 17β-estradiol; UHPLC-MS/MS, ultra-high-performance liquid chromatography–tandem mass spectrometry.

### 3.3 Evidence from human prostate tissue and human-derived models

Human prostate tissue and human-derived models provide an important translational bridge between population associations and animal experiments, but they support different levels of inference. BPA has been detected in histologically non-cancerous prostate tissue obtained from men with localized prostate cancer, demonstrating target-tissue accessibility but not an exposure–disease relationship or biological effect (Vitku et al., 2023). Primary human prostate epithelial cells also show broad transcriptional responses to low-nanomolar BPA exposure, including changes in nuclear-receptor and epigenetic-regulatory networks (Renaud et al., 2019). These findings demonstrate cellular responsiveness, but the acute exposure design does not establish persistent reprogramming or malignant transformation.

Stem/progenitor and developmental models further indicate that cellular state can influence BPA responsiveness. In primary human prostate stem/progenitor cultures, BPA increased prostasphere formation, expanded stem-like populations, and activated rapid protein kinase B (Akt)/extracellular signal-regulated kinase (ERK) signaling (Prins et al., 2014). In human embryonic stem cell-derived prostate organoids, BPA altered branching morphogenesis in a concentration-specific manner and increased focal accumulation of stem-like cells (Calderon-Gierszal and Prins, 2015). These models demonstrate BPA-responsive changes in self-renewal, signaling, and developmental organization, but they do not demonstrate neoplasia or reproduce intact human prostate carcinogenesis.

The most disease-proximal experimental evidence comes from a hybrid tissue-recombination model in which human prostate epithelial stem/progenitor cells were combined with embryonic rat urogenital sinus mesenchyme and grown in male nude mice. BPA exposure during graft morphogenesis—termed “developmental” exposure within this engineered model—increased the combined high-grade prostatic intraepithelial neoplasia (HG-PIN)/adenocarcinoma endpoint after a subsequent testosterone plus 17β-estradiol (T + E2) challenge (Prins et al., 2014). Interpretation is constrained by the human epithelial–rat mesenchymal–mouse host architecture, the experimental hormonal challenge, and the composite premalignant/malignant endpoint. Accordingly, this model supports altered susceptibility of human prostate epithelium under defined experimental conditions but does not demonstrate that BPA independently initiates human prostate cancer. Human prostate tissue and human-derived model evidence is summarized in Table 3.

**TABLE 3:** Human prostate tissue and human-derived models relevant to BPA exposure and susceptibility.

Study	Human source/model and donor characteristics	BPA exposure or assessment	Hormonal/model context	Endpoint and assessment	Result and statistical status	Evidence boundary
Vitku et al., 2023	Histologically non-cancerous prostate tissue from 20 men undergoing radical prostatectomy for localized PCa. Age and preoperative hormonal-treatment status: NR.	No experimental exposure; unconjugated and conjugated BPA measured in prostate tissue by LC–MS/MS; BPA LLOQ 1.571 pg/mg tissue	Clinical prostatectomy specimens; no experimental hormonal manipulation	Target-tissue BPA measurement; no exposure–outcome analysis	Unconjugated BPA detected in 35% and conjugated BPA in 60% of samples	Demonstrates prostate tissue accessibility. Detection in non-cancerous tissue from PCa-bearing prostates does not establish a biological effect, exposure–disease association, or causality
Renaud et al., 2019	Primary PrECs from one healthy 23-year-old male with no history of prostate disease; non-immortalized, TERT-negative cells. Donor hormonal status: NR.	5 or 25 nM BPA for 24 h; three biological microarray replicates from the same donor source	Primary epithelial culture; no defined androgen manipulation; EE2 used as an estrogenic comparator	Whole-genome transcriptomic profiling	5 and 25 nM BPA produced 7,182 and 7,650 differentially expressed transcripts, respectively (q ≤ 0.1)	Single-donor, acute-exposure study. Demonstrates transcriptional responsiveness, not persistent reprogramming, functional causality, or malignant transformation
Prins et al., 2014 — *in vitro* component	Primary human PrECs/prostaspheres from 4 disease-free organ donors aged 19–21 years. Donor hormonal status: NR.	0.1 nM–1 μM BPA for up to 7 days; rapid signaling examined at 10 nM	Primary-cell/Matrigel prostasphere system; no intact stromal or systemic environment	Prostasphere number/size, stem-like cell fraction, stemness markers, Akt/ERK signaling	Prostasphere number and size increased, with maximal effects at 10 nM; stem-like cell fraction increased and 10 nM BPA rapidly activated Akt and ERK; significant comparisons as reported	Demonstrates stem/progenitor responsiveness and altered self-renewal/signaling; these are surrogate cellular endpoints, not neoplastic transformation
Calderon-Gierszal and Prins, 2015	Engineered developmental model using H1/WA01 (46,XY) and H9 (46,XX) hESC lines; not primary prostate donor tissue. Donor age/disease state: N/A	1 or 10 nM BPA throughout directed differentiation; experiments replicated 6–9 times	Directed differentiation in Matrigel with defined growth factors and 1.7 μM testosterone	Branching morphogenesis, degenerating structures, differentiation/stem-cell markers	1 nM BPA increased budding (P < 0.001); 10 nM reduced budding (P < 0.05) and increased degenerating structures (P < 0.05); focal stem-like cell aggregates increased after BPA exposure	Engineered developmental model lacking maternal metabolism, systemic exposure, and the intact vascular/immune/endocrine environment. Morphogenesis and stem-cell changes are not PIN or cancer endpoints
Prins et al., 2014 — tissue-recombination component	Human prostate epithelial stem/progenitor cells derived from the young, disease-free donor PrEC source used in the study; donor number contributing to individual graft groups: NR. Human cells were combined with embryonic rat UGM and implanted into male nude mice	Oral BPA for 2 weeks at 100 or 250 μg/kg BW/day; additional 200 nM *in vitro* +250 μg/kg BW/day *in vivo* group. Free serum BPA after oral exposure: 0.39 and 1.35 ng/mL	After graft maturation, T + E2 treatment for 2–4 months	Combined HG-PIN/adenocarcinoma endpoint; sections coded and evaluated by two treatment-blinded board-certified pathologists	Combined HG-PIN/adenocarcinoma: 13% in vehicle versus 36%, 33%, and 45% in the three BPA groups; comparisons significant as reported	Hybrid human–rat–mouse susceptibility model requiring a later hormonal challenge. The composite premalignant/malignant endpoint does not demonstrate that BPA alone induces human prostate cancer

NR indicates information not reported in the main publication and should not be interpreted as evidence that the characteristic or procedure was absent. N/A indicates not applicable to the experimental model. The two entries from Prins et al. (2014) represent distinct experimental components within the same publication and are presented separately because they involve different model structures, exposure contexts, and endpoints. Detection of BPA in human prostate tissue indicates tissue accessibility but does not establish an exposure–disease association or causal effect.

Abbreviations: BPA, bisphenol A; BW, body weight; DE, differentially expressed; EE2, 17α-ethinylestradiol; hESC, human embryonic stem cell; HG-PIN, high-grade prostatic intraepithelial neoplasia; LC–MS/MS, liquid chromatography–tandem mass spectrometry; LLOQ, lower limit of quantification; N/A, not applicable; NR, not reported in the main article; PCa, prostate cancer; PrEC, primary human prostate epithelial cell; TERT, telomerase reverse transcriptase; T + E2, testosterone plus 17β-estradiol; UGM, urogenital sinus mesenchyme.

### 3.4 BPA substitutes and prostate-relevant evidence

Prostate-specific evidence for major BPA substitutes remains substantially smaller than the evidence base for BPA. Human data are limited primarily to target-tissue detection and cross-sectional or case–control biomonitoring rather than prospective disease studies. In histologically non-cancerous prostate tissue from men undergoing prostatectomy for localized PCa, conjugated BPS was detected in a minority of samples, whereas BPF was not detected; these measurements demonstrate possible target-tissue accessibility but do not establish comparative prostate-cancer risk (Vitku et al., 2023). In a multi-bisphenol case–control study, non-BPA bisphenols were detected infrequently, whereas BPA was the bisphenol associated with PCa in the reported regression analyses (Wang et al., 2024). Current human evidence is therefore insufficient to determine whether BPS or BPF confers lower, similar, or greater prostate-cancer risk than BPA.

Experimental comparisons nevertheless indicate that substitution does not necessarily eliminate prostate-relevant biological activity. In LNCaP and C4-2 cells, selected BPA analogues produced compound-specific disturbances in centrosome duplication and microtubule organization. BPS showed relatively strong activity in these assays, whereas BPF showed more limited acentrosomal effects. In the chronic soft-agar assay, BPS did not reproduce the BPA-associated increase in anchorage-independent growth; BPF was not included in that reported comparison, whereas DMBPA produced a BPA-like increase (Ho et al., 2017). At low nanomolar concentrations, BPA, BPS, and BPF also produced distinct transcriptomic responses in LNCaP and PC-3 cells. All three increased DNA-strand damage in the comet assay, whereas micronucleus formation was not increased, indicating DNA damage without evidence of stable chromosomal damage in that assay (Cortés-Ramírez et al., 2023). Additional BPS-specific studies reported increased proliferation and invasion of PC-3 and LNCaP cells together with increased COL1A1/COL1A2 expression (Liu et al., 2022), and increased proliferation of C4-2 and LNCaP cells at 10^−7^ M BPS (Ju et al., 2024). These functional observations support BPS responsiveness in prostate-cancer cell models; however, the accompanying database-derived risk models, target-prediction analyses, and molecular-docking results remain hypothesis-generating rather than evidence of human exposure effects or clinical progression. At higher micromolar concentrations, BPA, BPS, and BPF also produced partly overlapping, concentration- and cell-state-dependent stress and apoptotic responses in nonmalignant and malignant prostate-cell models (Caglayan and Ozden, 2024).

*In vivo* evidence for BPA substitutes remains limited and does not establish prostate carcinogenicity. In adult male mice, BPA, BPS, and BPF increased prostate mass and lower-urinary-tract phenotypes when co-administered with testosterone, whereas the bisphenols alone did not increase prostate mass and no prostate or urothelial cancer was observed (Nguyen et al., 2022). A recent comparative study of multiple bisphenols also reported low-concentration proliferative responses in prostate-cancer cells (Song et al., 2026), but its bioinformatic associations and molecular-docking analyses should not be interpreted as evidence of human bisphenol exposure or clinical progression. Overall, the available evidence argues against assuming that BPS or BPF are biologically inert substitutes for BPA, but it does not establish equivalent prostate carcinogenicity across bisphenols. The evidence is strongest for cellular activity, substantially weaker for *in vivo* prostate pathology, and currently inadequate for comparative human cancer-risk assessment.

## 4 Mechanistic framework for context-dependent modification of prostate susceptibility by BPA

Available evidence does not support a single, linear BPA-driven pathway to prostate carcinogenesis. BPA-associated effects have instead been reported across hormone-receptor and rapid-signaling, epigenetic, metabolic/redox, structural, and tissue-level domains under different experimental conditions. These response modules may interact, but most have been demonstrated in separate models rather than as a continuous causal sequence. The sections below therefore distinguish directly demonstrated BPA responses from disease-context evidence and integrative hypotheses.

### 4.1 Hormone-receptor and early signaling perturbation

BPA may alter prostate responsiveness through estrogen- and androgen-regulated signaling. Reported effects include rapid kinase activation, receptor-dependent transcription, and changes in systemic or local hormone availability. Their direction varies with dose, hormonal milieu, receptor genotype, and cell state. These effects are therefore considered context-dependent signaling responses rather than uniform carcinogenic mechanisms.

#### 4.1.1 Estrogen-responsive signaling and cellular plasticity

Estrogen-responsive signaling is an early interface through which BPA may alter prostate cell behavior, but the outcome depends on the broader receptor network. Estrogen receptor alpha (ERα) and estrogen receptor beta (ERβ) have distinct, compartment-specific roles that change with differentiation, aging, tumor progression, and AR signaling (Bonkhoff and Berges, 2009; Pellerin et al., 2021). Although BPA is estrogenic in receptor-responsive systems (Krishnan et al., 1993), prostate responses also depend on growth-factor signaling, AR status, and cellular plasticity.

In developmentally plastic cells, BPA may shift the balance between self-renewal and lineage commitment. Low-nanomolar BPA rapidly activated Akt and ERK in primary human prostate stem/progenitor cells and increased prostasphere formation and the stem-like fraction without classical estrogen-response-element (ERE)-dependent transcription (Prins et al., 2014). Phosphoinositide 3-kinase (PI3K)-Akt and mitogen-activated protein kinase (MAPK)-ERK regulate survival, proliferation, differentiation, and stem-cell behavior in prostate tissue (Rodríguez-Berriguete et al., 2012; Edlind and Hsieh, 2014; Di Donato et al., 2017; Shorning et al., 2020). These findings support altered cellular plasticity, not direct neoplastic transformation.

The same kinase network can produce different outcomes at other doses or in other receptor contexts. High-micromolar BPA activated an AR/ERβ-EGFR-ERK-p53 pathway, increased p21 and p27, and induced growth arrest in prostate epithelial and cancer cells (Bilancio et al., 2017). In contrast, low-nanomolar BPA supported a proliferation-associated transcriptional state and reduced ERβ expression in LNCaP cells carrying AR‐T877A (Hess-Wilson et al., 2007). ERK activation is therefore not inherently tumor-promoting. Its output depends on signal strength, duration, cell state, and AR context.

Estrogen-related receptor gamma (ERRγ) is a possible link between BPA recognition and metabolic state, but its prostate-specific role remains uncertain. BPA binds ERRγ with nanomolar affinity and stabilizes its constitutively active ligand-binding conformation (Takayanagi et al., 2006; Liu et al., 2007; Okada et al., 2008). In prostate cancer models, ERRγ restoration increased p21 and p27 and restrained proliferation and xenograft growth, whereas androgen-mediated ERRγ repression supported mitochondrial and lipid-metabolic remodeling (Yu et al., 2007; Audet-Walsh et al., 2017). These findings establish biological plausibility, not direct evidence that ERRγ mediates BPA responses in prostate cells.

Overall, BPA perturbs an interconnected ER-AR-growth-factor network rather than a single estrogen pathway. In plastic cells, this may alter self-renewal and differentiation; in selected mutant-AR settings, it may favor proliferation; and at higher concentrations, it may activate checkpoint responses. Estrogen-responsive signaling is therefore best viewed as a context-dependent regulator of prostate cell state and response thresholds.

#### 4.1.2 Androgen receptor state and hormonal context

BPA can influence androgen signaling at several levels, including systemic androgen production, intraprostatic steroid metabolism, and receptor-level signaling. Experimental studies have reported suppression of pituitary–testicular signaling and steroidogenic gene expression, whereas direct inhibition of CYP17A1 and 3β-hydroxysteroid dehydrogenase has generally been observed at higher BPA concentrations (Akingbemi et al., 2004; Ye L. et al., 2011). BPA also altered aromatase and 5α-reductase isoform expression in rat prostate, potentially shifting the local balance among testosterone, dihydrotestosterone (DHT), and estradiol (Castro et al., 2013; Sánchez et al., 2013). These findings support effects on hormone availability and local steroid metabolism but do not by themselves demonstrate tumor-promoting AR activation.

In wild-type or standardized AR systems, BPA has predominantly shown antagonistic or inhibitory effects on canonical receptor signaling. Reported effects include reduced androgen binding, disruption of AR–coregulator interactions, impaired nuclear localization, and decreased androgen-dependent transcriptional activity (Lee et al., 2003; Teng et al., 2013). Subsequent experimental studies identified impaired DHT-induced AR stabilization and altered AR–Hsp90 dynamics, consistent with a multistep antiandrogenic effect under the tested conditions (Teng et al., 2013; Huang et al., 2019). Primary human prostate epithelial cells also respond transcriptionally to low BPA concentrations: exposure to five or 25 nM BPA altered nuclear-receptor and androgen-responsive gene networks (Renaud et al., 2019). Because direct AR binding, nuclear translocation, and androgen-response-element activity were not tested in that study, these findings demonstrate prostate-cell responsiveness rather than direct AR agonism or antagonism.

The clearest evidence that receptor state can alter the direction of the BPA response comes from selected mutant-AR models. In androgen-depleted LNCaP cells carrying AR-T877A, low-nanomolar BPA promoted AR nuclear translocation, androgen-response-element binding, PSA expression, and proliferation (Wetherill et al., 2002). Subsequent work showed that these agonist-like responses depended on AR genotype, residual androgen, and BPA concentration: T877A and H874Y were permissive to BPA-associated activation, whereas wild-type AR was less responsive and higher BPA concentrations could inhibit growth (Wetherill et al., 2005). In castrated mice bearing AR‐T877A‐positive LNCaP xenografts, BPA was associated with earlier PSA recovery and continued tumor growth during androgen withdrawal (Wetherill et al., 2006). These findings support a receptor- and hormonal-context-dependent effect but remain specific to selected ligand-promiscuous mutant-AR systems.

This distinction is particularly important in advanced and castration-resistant prostate cancer, where AR signaling can evolve through amplification or overexpression, ligand-binding-domain mutations, and constitutively active splice variants such as AR-V7. Such alterations are enriched in advanced disease, and AR-V7 has been associated with reduced response to enzalutamide and abiraterone, while ligand-binding-domain mutations can alter antiandrogen pharmacology (Antonarakis et al., 2014; Robinson et al., 2015; Prekovic et al., 2016). These observations provide disease-state context rather than BPA-specific mechanisms: there is currently no direct evidence that BPA induces AR amplification, AR-V7 expression, or treatment-selected AR mutations.

Accordingly, BPA findings should be interpreted within the receptor and disease state of the model. Developmental or benign prostate systems, androgen-responsive and androgen-independent AR-positive cells, AR-null cells, mutant-AR models, and therapy-resistant states are not mechanistically interchangeable. AR-null models cannot establish canonical AR modulation, whereas responses in ligand-promiscuous mutant-AR models should not be generalized to wild-type AR disease. BPA should therefore not be characterized as uniformly androgenic or antiandrogenic; its reported effects depend on receptor genotype, hormonal milieu, cellular state, dose, and treatment context. Direct comparisons also indicate model-dependent effects: at higher BPA concentrations, growth inhibition was greater in AR-positive androgen-dependent LNCaP and LAPC-4 cells than in androgen-independent 22Rv-1 cells, whereas AR-negative cells showed little growth inhibition (Wetherill et al., 2005). However, direct BPA evidence in clinically evolved therapy-resistant models remains limited, and androgen-deprivation preconditioning should not be equated with resistance selected by enzalutamide or abiraterone.

### 4.2 Epigenetic regulation and persistence

Epigenetic alterations provide a plausible mechanism by which early BPA exposure could be associated with persistent changes in prostate cell state. However, prostate-specific effects are heterogeneous and vary with genomic locus, exposure window, dose, age, and cellular compartment, arguing against a uniform BPA-induced epigenetic program.

Developmental rodent studies provide the strongest evidence for persistence. Neonatal BPA exposure was associated with sustained PDE4D4 hypomethylation and increased expression during aging (Ho et al., 2006). Subsequent studies identified persistent or age-dependent methylation and expression changes at Nsbp1, Hpcal1, and additional loci (Tang et al., 2012; Cheong et al., 2016). These alterations varied with dose, age, and prostate lobe in models subsequently challenged with testosterone plus estradiol (Prins et al., 2017). In human prostate stem/progenitor-derived prostaspheres, BPA altered histone marks and suppressed several SNORD-family noncoding RNAs without broad changes in the measured CpG methylome (Ho et al., 2015). Gestational BPA exposure also altered Esr1 promoter methylation and expression together with Dnmt3a expression in fetal mouse prostate mesenchyme (Bhandari et al., 2019). Together, these findings support localized and context-dependent epigenetic responses rather than a shared methylation signature.

Importantly, persistence does not establish causality. Current prostate studies have not shown that individual BPA-associated methylation or histone changes are necessary or sufficient for later pathological phenotypes, and targeted reversal or rescue experiments are largely lacking. These epigenetic alterations should therefore be regarded as candidate mediators or molecular correlates of altered prostate cell state rather than established causal drivers of prostate carcinogenesis.

### 4.3 Metabolic and redox adaptation as an amplification network

Metabolic and redox responses represent additional BPA-responsive modules that may influence prostate-cell state, but direct causal links to earlier receptor or epigenetic changes have not been established. Because normal secretory epithelium and malignant prostate cells use distinct metabolic programs, the same disturbance may produce energetic injury in one state and adaptive plasticity in another. These responses should therefore be interpreted according to cellular and disease context rather than as a fixed downstream stage in a BPA-driven pathway.

#### 4.3.1 Metabolic plasticity

Prostate metabolism changes substantially with cellular and disease state. Normal secretory epithelium is specialized for citrate production, whereas malignant transformation is associated with greater citrate oxidation and increased metabolic flexibility (Costello and Franklin, 1991; 2000; 2005). BPA-associated metabolic changes should therefore not be interpreted as a uniform Warburg-like shift; their significance depends on whether the target is normal epithelium, an androgen-responsive tumor cell, or a metabolically adapted cancer cell.

BPA-specific evidence for metabolic perturbation remains limited and does not yet establish a consistent reprogrammed metabolic state. Chronic BPA exposure altered an HNF4α-centered transcriptional network involving mitochondrial and metabolic genes in adult rat prostate (Lam et al., 2016). More recently, BPA exposure in androgen-deprivation-preconditioned Tramp-C1 cells altered transcripts enriched for mitochondrial energy metabolism and increased ESR1, ESR2, and PFKFB4 expression (Zhou et al., 2025). However, that study did not directly demonstrate ESR1-dependent regulation of PFKFB4, measure the proposed downstream metabolic flux, or establish that PFKFB4 was necessary for the BPA-associated phenotype. The proposed BPA–ESR1–PFKFB4 axis should therefore be regarded as a mechanistic hypothesis rather than an established causal pathway. At higher BPA concentrations, reduced ATP and mitochondrial alterations accompanying apoptosis in RWPE-1 cells were more consistent with energetic injury than adaptive metabolic reprogramming (Liu et al., 2020).

Independent prostate-cancer studies provide important disease-state context for these findings. PFKFB4 contributes to glucose metabolism and redox adaptation in prostate cancer and is increased in androgen-independent LNCaP-derived cells (Ros et al., 2012; Li et al., 2020). Similarly, Xia et al. showed that androgen-independent LNCaP-AI cells had greater glucose uptake, lactate production, and glycolytic capacity than parental LNCaP cells, and identified a PRKAR2B–HIF-1α positive-feedback loop that promoted glycolysis and tumor growth (Xia et al., 2020). Genetic suppression of PRKAR2B reduced glycolytic activity in LNCaP-AI cells, whereas inhibition of glycolysis or HIF-1α substantially attenuated PRKAR2B-associated growth. These findings illustrate that androgen-independent adaptation can be accompanied by enhanced glycolytic dependence, but BPA was not tested in this study and there is no evidence that BPA activates the PRKAR2B–HIF-1α pathway.

Overall, metabolic plasticity provides a relevant disease-state context for interpreting BPA-associated changes, but a defined BPA-driven metabolic program has not been established. PFKFB4 remains a candidate convergence point between BPA-responsive transcription and prostate-cancer metabolism, and direct causal experiments are required to determine whether ESR1 or PFKFB4 is necessary for BPA-associated metabolic or tumor phenotypes.

#### 4.3.2 Oxidative and endoplasmic reticulum stress and parallel mechanisms

BPA can perturb redox and stress-response pathways, but the direction and biological significance of these responses depend strongly on dose and cellular state. In nonmalignant prostate epithelial models, micromolar BPA altered antioxidant capacity, glutathione homeostasis, and base-excision repair proteins and activated reactive oxygen species (ROS)-associated endoplasmic reticulum stress signaling through the EIF2AK3–ATF4–DDIT3/CHOP pathway (Kose et al., 2020; Caglayan and Ozden, 2024). Because these responses were accompanied by apoptosis, they are more consistent with stress-induced cellular injury than with a uniform tumor-promoting adaptation.

Mitochondrial quality control provides an important disease-state context for interpreting such stress responses. In prostate cancer models, Huang et al. showed that CDK12 inhibition increased mitochondrial ROS and impaired stress-induced mitophagy by disrupting FOXO3-dependent BNIP3 expression. Under enzalutamide treatment, CDK12 inhibition further increased mitochondrial dysfunction and sensitized prostate cancer cells to the drug, while combined CDK12 inhibition and enzalutamide suppressed tumor growth in xenograft models (Huang et al., 2026). In a paired metastatic castration-resistant prostate cancer (mCRPC) dataset, post-enzalutamide increases in CDK12/BNIP3 expression and mitophagy-pathway enrichment were also observed in several nonresponders. These findings indicate that mitochondrial stress and quality-control programs can contribute to therapy-stressed prostate cancer states. However, BPA was not tested, and there is currently no evidence that BPA regulates the CDK12–FOXO3–BNIP3 axis or reproduces this mechanism of enzalutamide resistance.

Other BPA-responsive pathways should be considered in parallel rather than as components of a single continuous stress cascade. Low-nanomolar BPA increased Orai1-dependent store-operated calcium entry and migration in prostate cancer cells, and inhibition of calcium entry reduced the migratory response (Derouiche et al., 2013). Because ROS and endoplasmic reticulum stress were not evaluated in that model, these findings do not establish a calcium–ROS–endoplasmic reticulum stress sequence. Similarly, low-dose BPA altered COX-2 and L-PGDS signaling in a rat prostate hyperplasia model, and inhibition of these pathways reduced the hyperplastic response (Wu et al., 2020). This finding supports prostaglandin signaling in a selected hyperplasia-related context but does not establish a persistent tumor-promoting inflammatory microenvironment.

Overall, BPA-associated oxidative/endoplasmic reticulum stress, calcium signaling, and inflammatory responses should be interpreted as context-dependent and partly independent modules. Mitochondrial stress adaptation is additionally relevant to therapy-resistant prostate cancer biology, but direct involvement of BPA in these quality-control mechanisms has not been established. Evidence from separate models should therefore not be integrated into an uninterrupted calcium–ROS–endoplasmic reticulum stress–inflammation–mitophagy pathway. Whether these response modules converge functionally in BPA-exposed human prostate tissue remains unresolved.

### 4.4 Structural and tissue-level phenotypes

Structural and tissue-level phenotypes provide downstream evidence that BPA can alter prostate cell organization, but these endpoints differ substantially in their relevance to carcinogenesis. In prostate-derived epithelial and cancer cells, low-nanomolar BPA increased centrosome amplification and disrupted centrosome duplication and microtubule organization; selected models also showed increased anchorage-independent growth (Tarapore et al., 2014; Ho et al., 2017). These findings indicate altered mitotic organization but do not establish persistent genomic instability or malignant transformation *in vivo*.

Developmentally plastic models further show that BPA can alter differentiation and tissue organization. BPA increased self-renewal in primary human prostate stem/progenitor cells and modified branching and lineage commitment in human embryonic stem cell-derived prostate organoids (Prins et al., 2014; Calderon-Gierszal and Prins, 2015). In a hybrid tissue-recombination model combining human prostate epithelium with rat mesenchyme in immunodeficient mice, BPA exposure during graft development followed by testosterone-plus-estradiol challenge increased the combined incidence of high-grade PIN and adenocarcinoma (Prins et al., 2014). Because this model incorporates nonhuman stromal and host components, a subsequent hormonal challenge, and a combined premalignant/malignant endpoint, it supports altered susceptibility rather than demonstrating BPA-induced human prostate cancer.

Stromal responses may provide an additional route to tissue remodeling. Prenatal BPA altered periductal stromal differentiation and reduced the proportion of stromal AR-positive cells, while other experimental models have reported BPA-associated fibroblast signaling, collagen deposition, and hyperplastic or epithelial–mesenchymal transition (EMT)-related remodeling (Ramos et al., 2001; Huang et al., 2018; He et al., 2024). However, these findings arise from different models and endpoints and have not been linked into a continuous causal sequence. Structural abnormalities, altered differentiation, and stromal remodeling should therefore be considered distinct tissue-level consequences of BPA exposure rather than established sequential steps toward prostate carcinogenesis.

### 4.5 Contextual determinants of divergent outcomes

Divergent findings across BPA studies are strongly influenced by exposure timing, internal exposure, and subsequent biological challenge. Developmental exposure occurs during tissue patterning and lineage specification, whereas adult exposure acts on a differentiated gland (Timms et al., 2005; Ogura et al., 2007). Route of administration can also alter internal exposure profiles; in neonatal rats, oral and subcutaneous BPA produced different serum profiles despite both modifying susceptibility to a later testosterone-plus-estradiol challenge (Prins et al., 2011). Administered dose alone should therefore not be treated as a complete measure of biologically relevant exposure.

The nature of the subsequent challenge is equally important. BPA alone did not produce a consistent prostate-pathology pattern in the CLARITY-BPA core study, whereas developmental exposure followed by adult testosterone plus estradiol increased selected PIN or tumor-related outcomes in a dose- and lobe-dependent manner (Ho et al., 2006; Prins et al., 2017; Prins et al., 2018; Camacho et al., 2019). By contrast, perinatal BPA did not enhance carcinogenesis in DMAB- or MNU-plus-testosterone-based models (Ichihara et al., 2003; Bosland et al., 2023). Adult background conditions also influenced reported phenotypes: chronic BPA exposure under a physiological testosterone background was associated with hyperplastic, preneoplastic, and inflammatory changes in Noble rats (Lam et al., 2016), whereas long-term oral BPA exposure in gerbils, with or without a high-fat diet, was associated with increased lesion burden and inflammation, although the small groups did not permit firm inference regarding BPA–diet interaction or synergy (Facina et al., 2018). Hormonal, genotoxic, and metabolic contexts are therefore not interchangeable.

Dose, receptor state, and cell identity further shape the direction and magnitude of BPA responses. Low-nanomolar BPA produced agonist-like responses in selected ligand-promiscuous mutant-AR models, whereas standardized wild-type AR systems predominantly showed antagonistic effects; higher concentrations in other prostate models were associated with checkpoint activation, apoptosis, or cellular injury (Wetherill et al., 2002; Wetherill et al., 2005; Bilancio et al., 2017; Huang et al., 2019; Song et al., 2023). Comparable low-nanomolar exposures can also produce different transcriptional responses across prostate cancer cell states (Cortés-Ramírez et al., 2023). Apparent non-monotonicity should therefore be evaluated within the same model and endpoint rather than inferred by comparing unrelated experiments.

Finally, the endpoint examined determines the strength of the disease inference. Transcriptional changes, prostasphere formation, centrosome abnormalities, hyperplasia, PIN, and carcinoma are not equivalent outcomes. Primary human cells and organoids establish cellular responsiveness and differentiation effects, tumor cell models define mechanisms within selected disease states, and animal studies test tissue pathology and persistence. Divergent findings across these systems are therefore better interpreted according to exposure window, internal exposure, later challenge, receptor and cellular state, and endpoint rather than as evidence for a single uniform BPA effect.

### 4.6 Integrated mechanistic interpretation

Taken together, the evidence supports a conditional network of BPA-responsive processes rather than a single BPA-driven carcinogenic pathway. BPA-specific studies have identified changes in hormone-receptor signaling, epigenetic regulation, metabolic and redox responses, calcium and inflammatory signaling, mitotic organization, differentiation, and stromal remodeling under defined experimental conditions. However, these effects were largely demonstrated in different models, exposure ranges, and biological states and have not been established as a continuous causal sequence within the same experimental system.

A testable working model is that BPA modifies the response of susceptible prostate states to subsequent biological challenges. Developmental exposure followed by testosterone-plus-estradiol challenge increased lesion-related outcomes in selected rodent and hybrid human epithelial/rat mesenchymal tissue-recombination models (Ho et al., 2006; Prins et al., 2014; Prins et al., 2017; Prins et al., 2018), whereas selected ligand-promiscuous mutant-AR models showed BPA-associated agonist-like signaling and continued tumor growth under androgen-limited conditions (Wetherill et al., 2002; Wetherill et al., 2005; Wetherill et al., 2006). These findings support developmental- and receptor-state-dependent modification of susceptibility, but they do not show that BPA alone is sufficient to initiate prostate cancer. Consistent with this distinction, BPA did not produce a consistent prostate-pathology pattern in the CLARITY-BPA core study and did not enhance carcinogenesis in an alternative MNU-plus-testosterone model (Camacho et al., 2019; Bosland et al., 2023).

The mechanistic links connecting early BPA responses to later pathology remain incompletely established. Persistent epigenetic changes, altered expression of metabolic genes such as PFKFB4, redox or stress responses, and structural phenotypes should therefore be regarded as candidate mediators unless their necessity or sufficiency is demonstrated experimentally. Independent prostate-cancer studies further show that glycolytic adaptation and mitochondrial quality-control programs can become important in androgen-independent or therapy-stressed disease states (Xia et al., 2020; Huang et al., 2026), but BPA was not tested in these pathways. Current evidence therefore supports biological plausibility and context-specific modification of prostate susceptibility rather than a continuous causal pathway from environmental BPA exposure to human prostate cancer initiation, progression, or treatment resistance. The major mechanistic modules and their evidential boundaries are summarized in Figure 2, whereas Figure 3 illustrates the divergence of BPA-associated responses across distinct experimental contexts.

**FIGURE 2:**
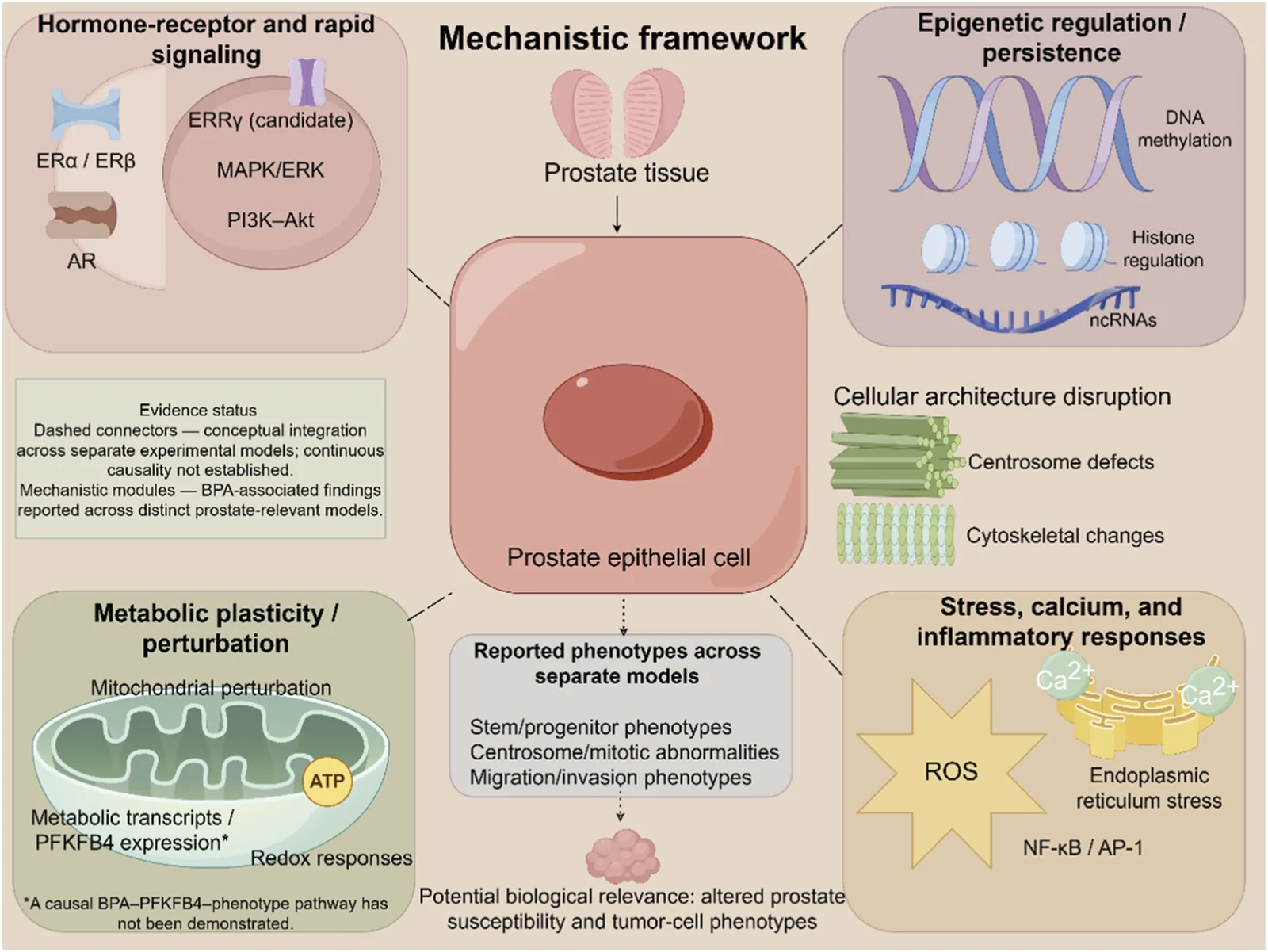
Integrated mechanistic framework of BPA-responsive processes relevant to prostate biology. The illustrated modules summarize BPA-associated findings reported across distinct prostate-relevant experimental systems and should not be interpreted as a continuous causal pathway. Dashed connectors indicate conceptual integration across separate experimental models rather than experimentally established sequential relationships. Epigenetic, metabolic, stress-related, structural, and cellular phenotypes are therefore presented as context-dependent responses or candidate mediators rather than established sequential steps toward prostate carcinogenesis. PFKFB4 represents a candidate metabolic node based on BPA-associated expression; a causal BPA–PFKFB4–phenotype pathway has not been demonstrated. Abbreviations: ERα, estrogen receptor alpha; ERβ, estrogen receptor beta; AR, androgen receptor; ERRγ, estrogen-related receptor gamma; MAPK, mitogen-activated protein kinase; ERK, extracellular signal-regulated kinase; PI3K, phosphoinositide 3-kinase; Akt, protein kinase B; ncRNAs, non-coding RNAs; ROS, reactive oxygen species; NF-κB, nuclear factor kappa B; AP-1, activator protein-1; ATP, adenosine triphosphate; Ca^2+^, calcium ion.

**FIGURE 3:**
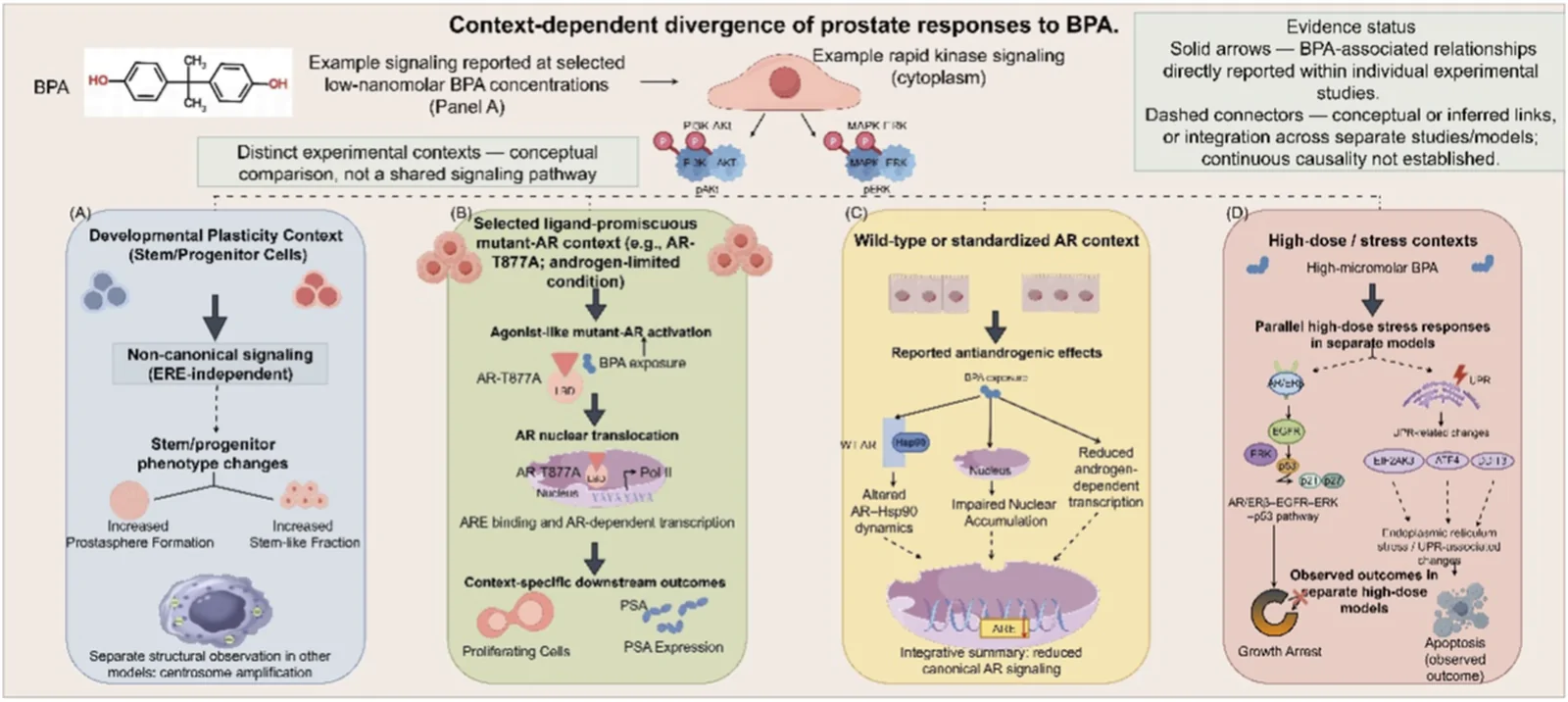
Context-dependent divergence of prostate responses to BPA. **(A–D)** depict distinct BPA-responsive experimental contexts **(A)** developmental or stem/progenitor responses **(B)** agonist-like signaling in selected ligand-promiscuous mutant-AR models **(C)** antiandrogenic responses in wild-type or standardized AR systems; and **(D)** growth-arrest or stress-related responses at higher concentrations. Solid arrows indicate directly reported BPA-associated relationships, whereas dashed connectors indicate conceptual or inferred relationships across separate studies or models. The illustrated contexts are not sequential and should not be interpreted as a continuous causal pathway to prostate carcinogenesis. Abbreviations: Akt, protein kinase B; AR, androgen receptor; ARE, androgen-response element; ATF4, activating transcription factor 4; BPA, bisphenol A; DDIT3/CHOP, DNA damage-inducible transcript 3/C/EBP homologous protein; EGFR, epidermal growth factor receptor; EIF2AK3/PERK, eukaryotic translation initiation factor 2-alpha kinase 3; ERβ, estrogen receptor beta; ERE, estrogen-response element; ERK, extracellular signal-regulated kinase; Hsp90, heat-shock protein 90; LBD, ligand-binding domain; MAPK, mitogen-activated protein kinase; PI3K, phosphoinositide 3-kinase; PSA, prostate-specific antigen; UPR, unfolded protein response.

## 5 Discussion: Translational perspectives and future directions

Although accumulating epidemiological and experimental evidence supports biological plausibility for BPA-associated effects on prostate biology, the clinical and public health implications remain incompletely defined. Rather than supporting immediate changes in screening, treatment, or regulatory practice, current evidence highlights the need to translate mechanistic and epidemiological findings into clinically meaningful strategies for risk assessment, precision prevention, and evidence-based policy. Accordingly, the following sections consider how current evidence may inform clinical practice, future research, and public health policy while acknowledging the substantial uncertainties that remain.

### 5.1 Implications for risk assessment, prevention, and early detection

At present, BPA exposure is not sufficiently validated for individual-level prostate cancer risk assessment. Human studies have reported heterogeneous associations between BPA exposure and prostate cancer-related outcomes, while most biomonitoring studies rely on a single serum or urinary measurement despite substantial within-person variability in BPA concentrations (Ye X. et al., 2011; Tarapore et al., 2014; Morgan et al., 2018; Salamanca-Fernández et al., 2021; Álvarez-González et al., 2024; Wang et al., 2024). Repeated or appropriately pooled samples may improve exposure classification (Philippat and Calafat, 2021), but clinically interpretable exposure ranges and incremental predictive value beyond established risk factors have not been established.

The reported cross-sectional association between urinary BPA and serum PSA should also be interpreted cautiously. PSA is influenced by malignant disease, benign prostatic enlargement, inflammation, and other prostatic or systemic factors; therefore, a BPA–PSA association does not demonstrate prostate cancer initiation or identify a validated cancer biomarker (Li et al., 2024). Current evidence does not support using bisphenol exposure histories or biomonitoring results to modify PSA thresholds, screening intervals, biopsy criteria, or individual risk classification.

A further translational limitation is the gap between experimental exposure conditions and human internal exposure. Nominal concentrations used in cell culture and administered doses in animal studies cannot be directly equated with BPA concentrations measured in human biological matrices. Experimental studies identify developmental exposure as a candidate susceptibility window and demonstrate persistent molecular or tissue responses in selected hormonal contexts, but they do not provide quantitative estimates of adult human risk or define a clinically identifiable high-risk subgroup (Ho et al., 2006; Prins et al., 2014; Prins et al., 2017; Prins et al., 2018). Although short-term interventions indicate that some sources of recent BPA exposure are modifiable, no study has shown that exposure reduction lowers prostate cancer incidence or improves screening outcomes (Carwile et al., 2011; Peng et al., 2019). Exposure reduction should therefore be considered a general precautionary strategy rather than a validated prostate-cancer-specific preventive intervention.

### 5.2 Implications for treatment response and precision medicine

Current evidence does not establish BPA exposure as a determinant of prostate cancer treatment response. The limited post-diagnosis human evidence consists primarily of an exploratory association between urinary BPA and biochemical recurrence after radical prostatectomy (Zhou et al., 2025). This finding is relevant to disease progression or prognosis, but it does not demonstrate an effect on response to androgen-deprivation therapy (ADT), androgen-receptor pathway inhibitors, or other systemic treatments. Experimental studies provide a more specific but model-dependent signal: in prostate cancer models carrying the ligand-promiscuous AR‐T877A variant, BPA maintained AR signaling and proliferative or tumor-growth responses under androgen-limited conditions (Wetherill et al., 2002; Wetherill et al., 2005; Wetherill et al., 2006). These observations support receptor-state-dependent responsiveness to BPA, but they do not establish that BPA causes clinical resistance to ADT or contemporary AR-targeted therapies.

Clinical translation will therefore require a clear distinction between prognostic associations and predictive exposure–treatment interactions. Future studies should assess repeated bisphenol exposure before and during defined therapies and integrate exposure data with treatment line, tumor AR status, hormonal context, and prespecified clinical outcomes. AR copy-number gain and ligand-binding-domain mutations are established features of treatment-evolved prostate cancer and have been associated with poorer outcomes during abiraterone treatment (Romanel et al., 2015); however, current evidence does not show that BPA induces these alterations. Analyses should also account for renal function, diet, medication use, and relevant co-exposures. Until reproducible exposure–treatment interactions are demonstrated, bisphenol measurements should not guide ADT or AR-inhibitor selection, treatment sequencing, monitoring intensity, or additional molecular testing.

### 5.3 Public health and regulatory implications

Public health and regulatory actions concerning BPA are based on evidence across multiple toxicological endpoints and should not be interpreted as confirmation of a prostate-cancer-specific hazard. In 2022, the California Office of Environmental Health Hazard Assessment (OEHHA) initiated a formal review of BPA carcinogenicity for possible listing under Proposition 65 (California Environmental Protection Agency Office of Environmental Health Hazard Assessment, 2022). At the subsequent Carcinogen Identification Committee meeting, however, the committee declined to list BPA as a carcinogen by a 5–6 vote (California Environmental Protection Agency Office of Environmental Health Hazard Assessment, 2023).

This outcome illustrates that regulatory hazard evaluation and prostate-cancer-specific causal inference are distinct questions. Separately, the European Food Safety Authority (EFSA) substantially lowered the tolerable daily intake for BPA in its 2023 re-evaluation, with immune-system effects forming the principal basis of the assessment, and concluded that estimated dietary exposure raised a public health concern (EFSA Panel on Food Contact Materials et al., 2023). The European Union subsequently adopted Commission Regulation (EU) 2024/3190, prohibiting BPA in specified food-contact materials subject to defined derogations and extending controls to other hazardous bisphenols and bisphenol derivatives (European Commission, 2024). These measures reflect broader toxicological and population-exposure evidence rather than proof that BPA causes human prostate cancer.

Substitution creates a related policy challenge. As discussed in Section 3.4, BPS, BPF, and other BPA analogues can retain endocrine-disrupting and prostate-relevant biological activity, but current evidence does not establish either their biological safety or equivalent prostate carcinogenicity (Rochester and Bolden, 2015; Le Fol et al., 2017; Lin et al., 2021). Regulatory strategies should therefore combine class-informed screening with compound-specific hazard assessment, product surveillance, and repeated population biomonitoring to determine whether BPA restrictions reduce aggregate bisphenol exposure rather than redistribute exposure among analogues (Kassotis et al., 2020). For prostate cancer specifically, current policy relevance lies in reducing avoidable exposure and strengthening post-regulatory evidence; disease-specific warnings, screening modifications, or prevention claims are not presently supported. Figure 4 summarizes these potential intervention points across source governance, exposure management, and clinical awareness. Its clinical components should be interpreted as areas for future evaluation rather than validated prostate-cancer-specific recommendations.

**FIGURE 4:**
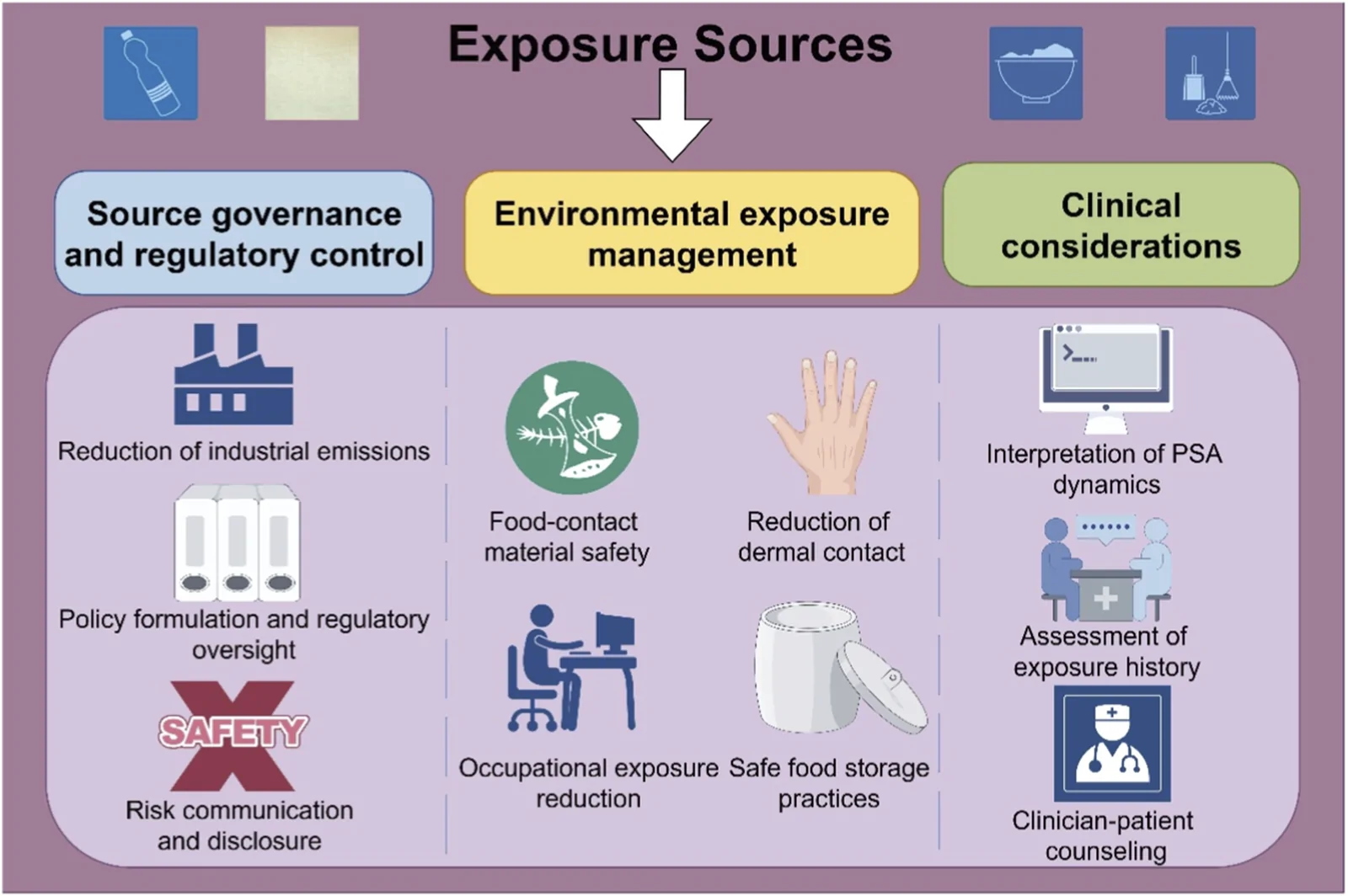
Conceptual framework for bisphenol exposure governance and public health translation. This schematic extends the policy discussion by illustrating potential intervention points across source governance and regulatory control, environmental exposure management, and clinical awareness. Regulatory and environmental components include industrial-emission control, product and food-contact material safety, occupational and dermal exposure reduction, and risk communication. The clinical components are presented as areas for future contextual evaluation rather than validated prostate-cancer-specific recommendations; current evidence does not support exposure-based modification of PSA interpretation, screening, or treatment. Abbreviations: PSA, prostate-specific antigen.

### 5.4 Research priorities for causal and translational validation

Future research should integrate exposure measurement, biological mechanism, and clinically relevant outcomes rather than advancing these domains independently. Human studies should prioritize repeated pre-diagnostic biomonitoring because urinary BPA concentrations show substantial within-person variability and a single measurement may poorly represent longer-term exposure (Ye X. et al., 2011; Morgan et al., 2018). Exposure assessment should use harmonized analytical and quality-control procedures and extend to major BPA analogues and relevant co-exposures within a class-informed framework (Kassotis et al., 2020). Longitudinal cohorts using serial or appropriately timed archived biospecimens should preserve the temporal ordering between exposure and outcome and distinguish etiologic, prognostic, and predictive questions (Salamanca-Fernández et al., 2021). Cross-cohort replication, transparent reporting of detection limits and exposure handling, and harmonized clinical endpoints will be essential for determining whether reported associations are reproducible and clinically informative.

Experimental research should move from cataloguing BPA-responsive pathways toward testing causality and human relevance. Primary human prostate cells, organoids, and tissue-based models demonstrate biological responsiveness to BPA but represent selected developmental, epithelial, and hormonal contexts rather than the intact human prostate (Prins et al., 2014; Calderon-Gierszal and Prins, 2015; Renaud et al., 2019). Future studies should compare developmental, benign, treatment-naïve, and therapy-resistant states using exposure conditions anchored, where possible, to measured human internal concentrations and should incorporate stromal, immune, and endocrine components. Dose–response relationships, including potential non-monotonic responses, should be tested within the same model and endpoint rather than inferred across heterogeneous experiments. Genetic or pharmacological perturbation should establish whether candidate receptor, epigenetic, metabolic, redox, and structural changes are necessary for persistent phenotypes rather than merely associated with them.

Independent replication across laboratories and complementary experimental systems is particularly important because hormone-challenge models, unchallenged studies, and alternative carcinogenesis paradigms have produced divergent prostate outcomes (Prins et al., 2018; Camacho et al., 2019; Bosland et al., 2023). Species differences, exposure route and internal dose, later biological challenge, and pathological criteria should therefore be incorporated explicitly into cross-study interpretation, and null or discordant findings should be reported alongside positive results. Translation should proceed sequentially from reproducible association and causal mechanistic validation to improvement in risk or treatment prediction, demonstration of clinical utility, and only thereafter consideration of prostate-cancer-specific clinical guidance or regulatory action.

### 5.5 Limitations of the current evidence base

A major limitation of the current literature is not simply the small number or heterogeneity of individual studies, but the lack of direct continuity across evidence domains. Human studies examine endpoints ranging from prostate cancer diagnosis and biochemical recurrence to PSA concentrations. In contrast, experimental studies evaluate outcomes such as receptor activation, transcriptional responses, stem/progenitor behavior, PIN, or challenge-dependent tumor phenotypes. These endpoints differ in biological meaning and cannot be arranged as sequential stages of a single causal pathway. Consequently, mechanistic plausibility cannot by itself validate an epidemiological association, and an experimental prostate phenotype cannot be assumed to predict human cancer risk.

Exposure metrics are similarly non-equivalent across study types. Urinary or serum biomonitoring, target-tissue measurements, nominal cell-culture concentrations, and administered animal doses represent different dimensions of exposure and cannot be directly ranked on a common scale. This problem is compounded by temporal mismatch: much of the human evidence reflects exposure measured near diagnosis or after disease has developed, whereas some of the strongest experimental findings arise from early-life exposure followed by a later hormonal challenge. Thus, correspondence in biological response does not establish correspondence in exposure history or disease causation.

Additional limitations arise from selective reporting and incomplete methodological harmonization across the literature. Publication bias and preferential emphasis on positive findings cannot be excluded, while exposure assessment varies substantially in biological matrix, sampling time, normalization strategy, detection limits, exposure route, and administered dose. Experimental systems also differ in species and strain, prostate region, developmental window, hormonal background, receptor state, and pathological criteria, and dose selection is not consistently anchored to measured internal exposure. Potential non-monotonic responses further complicate interpretation and should be evaluated within the same model and endpoint rather than inferred across heterogeneous experiments. Finally, several influential findings derive from closely related experimental paradigms, and independent replication across laboratories and complementary model systems remains limited.

These discontinuities define the principal unresolved translational problem. Current evidence supports the prostate’s biological responsiveness to BPA in selected settings. However, it does not yet connect a defined human exposure trajectory to a validated intermediate mechanism and, subsequently, to a clinically verified prostate cancer outcome. Establishing that chain—while showing that the same relationship is reproducible across populations and human-relevant models—remains necessary before causal, predictive, or preventive conclusions can be justified.

## 6 Conclusion

Current evidence does not establish BPA as a human prostate carcinogen, but it does show that prostate responses to BPA vary substantially with exposure timing, internal dose, hormonal environment, receptor status, cellular state, and subsequent biological challenge. Human studies provide limited and heterogeneous associations, whereas experimental models demonstrate biological responsiveness and identify candidate hormonal, epigenetic, metabolic, redox, and structural mechanisms. These evidence domains strengthen biological plausibility but do not yet form a continuous causal chain from human exposure to prostate cancer.

Three questions remain central. First, does a defined pattern of BPA exposure preceding disease onset increase the risk of clinically verified prostate cancer? Second, which BPA-responsive molecular or cellular changes are necessary mediators of persistent prostate phenotypes rather than associated responses? Third, do hormonal state, receptor genotype, developmental timing, or other host factors identify reproducible subgroups in which BPA meaningfully alters disease susceptibility or progression? Until these questions are resolved, bisphenol measurements should not be used for prostate cancer screening, prognostic assessment, or treatment decisions. Future studies should therefore prioritize longitudinal exposure assessment, causal validation in human-relevant models, clinically verified outcomes, and compound-specific evaluation of BPA substitutes within a broader class-informed framework.
